# The Optimal Cut-Off Value of Neutrophil-to-Lymphocyte Ratio for Predicting Prognosis in Adult Patients with Henoch–Schönlein Purpura

**DOI:** 10.1371/journal.pone.0153238

**Published:** 2016-04-13

**Authors:** Chan Hyuk Park, Dong Soo Han, Jae Yoon Jeong, Chang Soo Eun, Kyo-Sang Yoo, Yong Cheol Jeon, Joo Hyun Sohn

**Affiliations:** Department of Internal Medicine, Hanyang University Guri Hospital, Hanyang University College of Medicine, Guri, Korea; Renal Division, Peking University First Hospital, CHINA

## Abstract

**Background:**

The development of gastrointestinal (GI) bleeding and end-stage renal disease (ESRD) can be a concern in the management of Henoch–Schönlein purpura (HSP). We aimed to evaluate whether the neutrophil-to-lymphocyte ratio (NLR) is associated with the prognosis of adult patients with HSP.

**Methods:**

Clinical data including the NLR of adult patients with HSP were retrospectively analyzed. Patients were classified into three groups as follows: (a) simple recovery, (b) wax & wane without GI bleeding, and (c) development of GI bleeding. The optimal cut-off value was determined using a receiver operating characteristics curve and the Youden index.

**Results:**

A total of 66 adult patients were enrolled. The NLR was higher in the GI bleeding group than in the simple recovery or wax & wane group (simple recovery vs. wax & wane vs. GI bleeding; median [IQR], 2.32 [1.61–3.11] vs. 3.18 [2.16–3.71] vs. 7.52 [4.91–10.23], *P*<0.001). For the purpose of predicting simple recovery, the optimal cut-off value of NLR was 3.18, and the sensitivity and specificity were 74.1% and 75.0%, respectively. For predicting development of GI bleeding, the optimal cut-off value was 3.90 and the sensitivity and specificity were 87.5% and 88.6%, respectively.

**Conclusions:**

The NLR is useful for predicting development of GI bleeding as well as simple recovery without symptom relapse. Two different cut-off values of NLR, 3.18 for predicting an easy recovery without symptom relapse and 3.90 for predicting GI bleeding can be used in adult patients with HSP.

## Introduction

Henoch–Schönlein purpura (HSP) is a leukocytoclastic vasculitis with immunoglobulin A (IgA) deposition [1–3]. It typically involves the small vessels of the skin, gastrointestinal (GI) tract, joints, and kidneys [4]. Although the prognosis of HSP is generally good, the development of potentially life-threatening conditions, including GI bleeding and end-stage renal disease (ESRD), can be a concern in the management of HSP [2,5,6]. Because it has been suggested that early corticosteroid administration might be helpful in improving clinical outcomes, including symptom relief and risk of surgical intervention [7–9], a reliable prognostic marker is required to select patients who should be treated with corticosteroid.

Several laboratory tests, including the neutrophil-to-lymphocyte ratio (NLR), mean platelet volume, D-dimer, and fibrinogen degradation product, have been suggested as prognostic markers in children with HSP [10–13]. However, laboratory markers for predicting poor prognosis in adult patients have been poorly studied. Because the clinical manifestation and disease course of HSP differ between adults and children, prognostic markers for adult patients should be evaluated separately. Adult patients with HSP often show more serious GI bleeding, requiring red blood cell (RBC) transfusion or surgery, compared to children [1–3]. Additionally, renal involvement of HSP and the development of chronic kidney disease are more common in adults than in children [14,15]. Therefore, there is a greater need for a reliable prognostic marker of HSP in adult patients than in pediatric patients.

Among the prognostic markers in children with HSP, we were interested in NLR, a measurement of systemic inflammation, because it is also a well-known risk factor of various adult diseases, including cardiovascular disease, liver cirrhosis, various cancers, and inflammation in ESRD patients [16–19]. If the development of potentially life-threatening disease, including GI bleeding and ESRD, is associated with a severe immune response, the NLR may also be associated with the poor prognosis of patients with HSP. Here, we evaluated whether the NLR is associated with the prognosis of adult patients with HSP. Furthermore, we aimed to define the optimal cut-off values of NLR for predicting disease course for clinical use.

## Methods

We retrospectively reviewed the clinical records of patients who were diagnosed with HSP at Hanyang University Guri Hospital, Guri, Korea, between June 2004 and August 2015. Patients were assessed by the criteria of Michel *et al*. [3] Using the Michel’s method, patients who met three or more following criteria were diagnosed with HSP: (1) palpable purpura, (2) bowel angina, (3) GI bleeding, (4) hematuria, and (5) absence of medication. Patients who were younger than 18 years were excluded. Patients with immunologic disorders were also excluded because the disease or medication can affect NLR regardless of severity of HSP. We collected the following data from patient medical records: demographics, comorbidities, presenting symptoms, initial laboratory data, and treatment outcomes. The NLR was calculated based on the results of a complete blood count test (CBC) performed during the first visit to the hospital. All blood samples were tested within 30 minutes in the laboratory of our institution. Any treatment for patients with HSP had started after blood sampling for laboratory tests including CBC. Institutional Review Board on Human Subjects Research and Ethics Committees of Hanyang University Guri Hospital approved this study. Patient records/information was anonymized and de-identified prior to analysis.

### Assessment of gastrointestinal bleeding and renal involvement

When patients showed clinical signs of GI bleeding, including hematemesis, melena, and hematochezia, esophagogastroduodenoscopy (EGD) and colonoscopy were performed to identify the source of bleeding. Mucosal biopsies were performed when deemed necessary by the endoscopist. Histological findings of small vessel vasculitis with polymorphonuclear leukocyte infiltration (leukocytoclastic vasculitis) were regarded as the characteristic pathologic findings of HSP. Additionally, either an abdominal CT scan or ultrasonography was performed on patients with GI bleeding and abdominal pain in order to assess the cause of GI bleeding or abdominal pain.

In addition, all patients were tested via urinalysis and serum creatinine to assess the renal involvement of HSP. When patients showed hematuria (>5 RBCs per high power microscopic field in a centrifuged specimen) or proteinuria (>300 mg/24 hours), a renal biopsy was recommended for further evaluation. In immunofluorescence assays, the predominance of mesangial IgA among glomerular immunoglobulin deposits was regarded as the pathologic finding of renal involvement of HSP.

### Treatment method and outcomes

Supportive care including resting and intravenous hydration was a mainstay of treatment in patients with HSP. However, some patients who showed severe abdominal pain, massive GI bleeding, or severe hematuria or proteinuria were treated with oral corticosteroid or steroid pulse therapy at the discretion of the clinician.

The disease courses of patients were classified into three groups according to symptom relief and relapse and development of GI bleeding, as follows: (a) simple recovery, (b) wax & wane without GI bleeding, and (c) development of GI bleeding. Wax & wane was defined as the relapse of any HSP-related symptoms within 3 months from the initial recovery.

### Statistical analysis

Continuous variables, including age, are presented as mean with standard deviation (SD). However, laboratory data including white blood cell (WBC), hemoglobin, platelet, and NLR are presented as median with interquartile range (IQR), because these data are not usually assumed to follow a normal distribution. Categorical variables were presented as sample number with proportion. The NLR according to disease course was compared by Mann-Whitney U test or Kruskal-Walis test. In order to assess the predictive performance of the NLR with respect to disease course, two receiver operating characteristics (ROC) curves were plotted. One was a ROC curve for predicting simple recovery of patients, and the other was that for predicting development of GI bleeding. Subsequently, two cut-off values of NLR were determined based on the Youden index from each ROC curve. Additionally, ROC curves of three other laboratory markers, including mean platelet volume (MPV), erythrocyte sedimentation rate (ESR), and C-reactive protein (CRP), were plotted. All statistical analyses were performed using the statistical software program SPSS for Windows (version 18.0; SPSS Inc., Chicago, IL, USA).

## Results

### Baseline characteristics

Among the 145 patients who were diagnosed with HSP, 83 who were younger than 18 years were excluded. In addition, one patient with Crohn’s disease taking mesalazine was excluded. As a result, 61 patients with HSP were retrospectively analyzed in the study. All patients were followed until full recovery or death. The median follow-up duration of patients was 34 days (IQR, 15–87 days). The mean age was 47 years, and 55.7% of patients were men (Table 1). Palpable purpura was shown in all patients. Twenty (32.8%) and 15 (24.6%) patients had diffuse abdominal pain and arthralgia, respectively.

**Table 1:** Baseline characteristics of the enrolled patients.

Variable	Value
N	61
Age, year, mean ± SD	47.0 ± 19.7
Sex, n (%)	
Male	34 (55.7)
Female	27 (44.3)
Comorbidity, n (%)	
Hypertension	13 (21.3)
Diabetes	10 (16.4)
Cerebrovascular disease	2 (3.3)
Cirrhosis	2 (3.3)
ESRD	2 (3.3)
Presenting symptom, n (%)	
Palpable purpura	61 (100.0)
Diffuse abdominal pain	20 (32.8)
Arthralgia	15 (24.6)

ESRD, end-stage renal disease; SD, standard deviation

### Laboratory tests and disease course

The results of the demographics, initial laboratory tests, treatment methods, and disease courses according to the GI bleeding are presented in Table 2. Of the 61 patients, 17 (27.9%) showed GI bleeding. In patients with GI bleeding, the median follow-up duration was 62 days (IQR, 26–128 days), while that of patients without GI bleeding was 31 days (IQR, 13–69 days). The medians of WBC, neutrophil count, and NLR were higher in the GI bleeding group than in the non-GI bleeding group (median [IQR]: WBC, 14,300 [10,950–17,600] vs. 7,500 [5,875–9,825], *P*<0.001; neutrophil, 12,488 [8,359–15,089] vs. 4,644 [3,510–6,825], *P*<0.001; NLR, 7.52 [4.91–10.23] vs. 2.53 [1.75–3.27], *P*<0.001). In contrast, the median of lymphocyte was lower in the GI bleeding group than in the non-GI bleeding group (median [IQR]: 1,430 [1,249–2,7] vs. 1,867 [1,456–2,441], *P* = 0.048). Proteinuria was more common in the GI bleeding group than in the non-GI bleeding group (52.9% vs. 18.2%, *P* = 0.010).

**Table 2:** Demographics, laboratory findings, and clinical course of the patients according to the gastrointestinal bleeding.

Variable	Non-GI bleeding	GI bleeding	*P*-value
N	44	17	
Demographics			
Age, year, median (IQR)	48.5 (33.3–64.0)	42.0 (19.5–64.0)	0.318
Male, n (%)	23 (47.7)	6 (35.3)	0.381
Initial laboratory finding			
Serum, median (IQR)			
WBC, /mm^3^	7,500 (5,875–9,825)	14,300 (10,950–17,600)	< 0.001
Hemoglobin, g/dL	13.6 (12.6–14.7)	14.6 (12.5–16.3)	0.348
Platelet, /mm^3^	245,000 (203,000–292,000)	244,000 (192,000–340,500)	0.641
MPV, fl	8.0 (7.3–8.7)	7.6 (7.3–9.2)	0.833
Neutrophil, /mm^3^	4,644 (3,510–6,828)	12,488 (8,359–15,089)	< 0.001
Lymphocyte, /mm3	1,867 (1,456–2,441)	1,430 (1,249–2,007)	0.048
NLR	2.53 (1.75–3.27)	7.52 (4.91–10.23)	< 0.001
PT,^a^ INR	0.96 (0.91–1.02)	1.03 (0.93–1.12)	0.077
aPTT,^a^ sec	32 (29–35)	30 (27–34)	0.155
ESR,^a^ mm/hr	22 (13–40)	15 (7–54)	0.628
CRP,^a^ mg/dL	0.61 (0.16–1.27)	2.42 (0.69–4.67)	0.006
Urine, n (%)			
Hemautria	16 (36.4)	8 (47.1)	0.443
Proteinuria	8 (18.2)	9 (52.9)	0.011
Treatment			0.010
Supportive care	23 (52.3)	7 (41.2)	
Oral corticosteroid	21 (47.7)	6 (35.3)	
Steroid pulse therapy	0 (0.0)	4 (23.5)	
Disease course and complication, n (%)			
Wax and wane	9 (20.5)	4 (23.5)	> 0.999
Development of ESRD	0 (0.0)	2 (11.8)	0.074
Death	0 (0.0)	1 (5.9)	0.279

^a^Some data were unavailable in 12, 13, and 8 patients for PT and aPTT, ESR, and CRP, respectively.

WBC, white blood cell; MPV, mean platelet volume; NLR, neutrophil-to-lymphocyte ratio; PT, prothrombin time; INR, international normalized ratio; aPTT, activated partial thromboplastin time; ESR, erythrocyte sedimentation rate; CRP, C-reactive protein; GI, gastrointestinal; ESRD, end-stage renal disease; IQR, interquartile range; N/A, not available

In the GI bleeding group, 7 (41.2%), 6 (35.3%), and 4 (23.5%) patients were treated with supportive care, oral corticosteroid, and steroid pulse therapy, respectively. In the four patients taking the steroid pulse therapy, one was identified as having hematuria, one had proteinuria, and two had both conditions in the initial urinalysis. Among the patients with GI bleeding, the renal function of two patients progressed to ESRD on 10 and 35 days from the admission, respectively, despite steroid pulse therapy. Moreover, one patient who showed both GI bleeding and ESRD died from uncontrolled GI bleeding. On the contrary, ESRD did not develop in any patient without GI bleeding.

In total, 57.4% of patients recovered without symptom relapse, and 14.8% of patients showed a wax and wane disease course but were ultimately cured without any life-threatening disease, including GI bleeding or ESRD. The remaining 27.9% of patients showed GI bleeding with or without development of ESRD. In cases of GI bleeding, only 41.2% of patients (7 of 17) showed clinical symptoms and signs associated with GI bleeding, including hematemesis, melena, and hematochezia, at the time of their hospital visit. In other words, the remaining 58.8% of patients who showed GI bleeding had no specific symptoms or signs of GI bleeding during the initial visit to the hospital. Five of seven (71.4%) patients who showed GI bleeding before visiting the hospital were treated with steroid therapy, while 5 of 10 (50.0%) who showed GI bleeding after visiting the hospital received steroid therapy.

**Fig 1:**
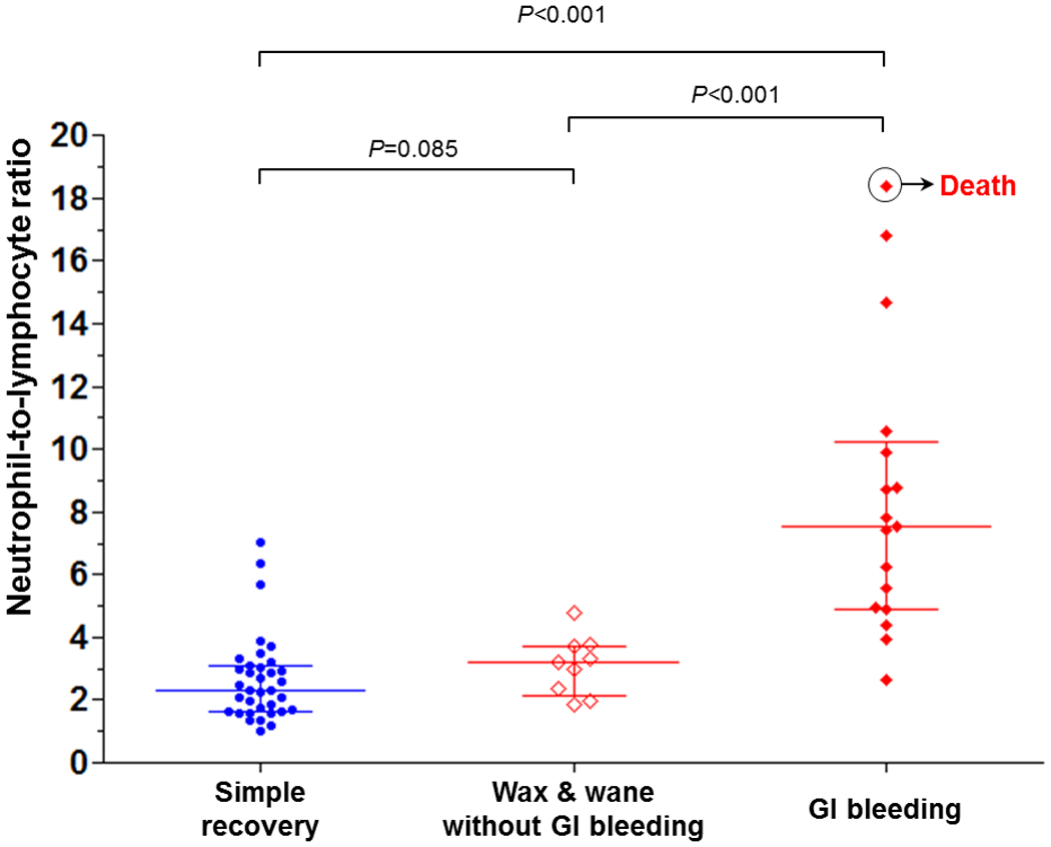
Initial neutrophil-to-lymphocyte ratio of patients according to disease course. Bar represents median and interquartile range. GI, gastrointestinal.

The scatter plot of NLR according to disease course is presented in Fig 1. The NLR was higher in the GI bleeding group than in the simple recovery or the wax & wane group (simple recovery vs. wax & wane vs. GI bleeding; median [IQR], 2.32 [1.61–3.11] vs. 3.18 [2.16–3.71] vs. 7.52 [4.91–10.23], *P* < 0.001).

### Optimal cut-off value of NLR

**Fig 2:**
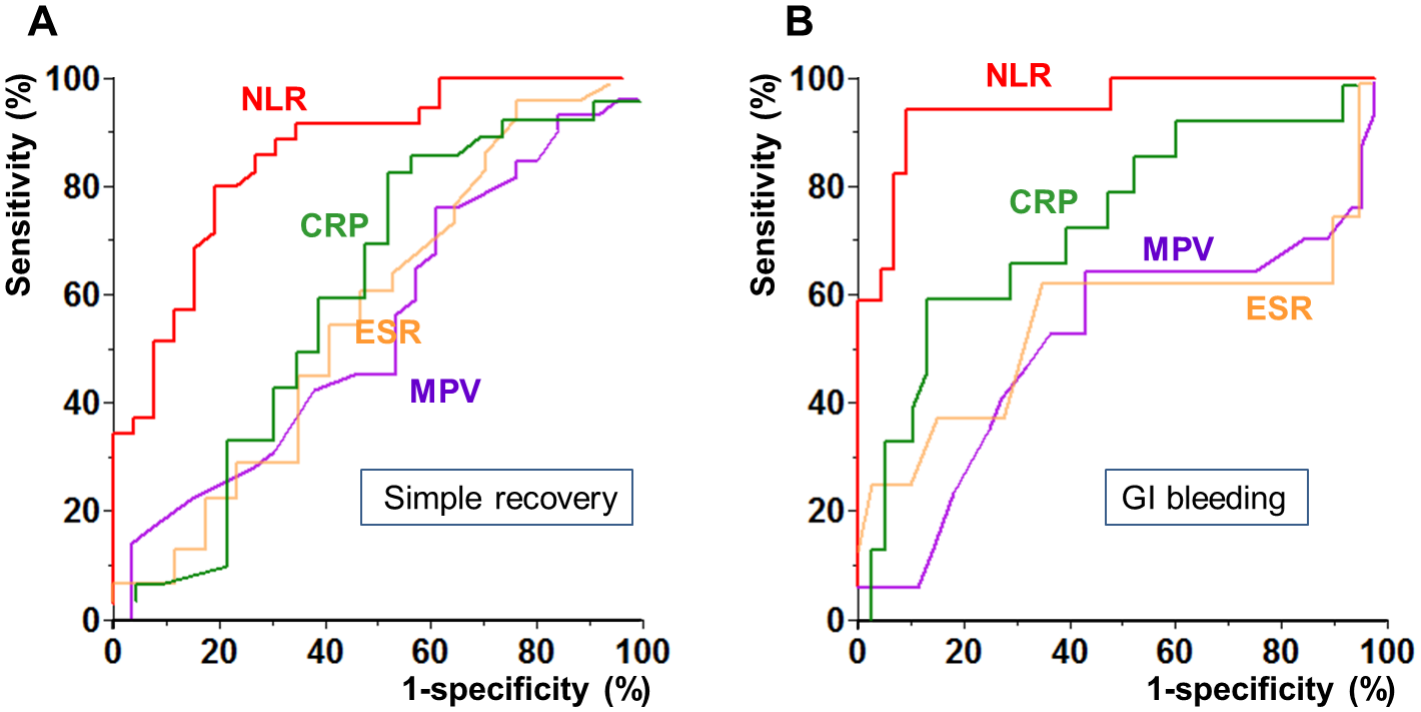
Receiver operating characteristics curves for predicting simple recovery (A) and development of GI bleeding (B). GI, gastrointestinal.

Fig 2 shows two ROC curves of four laboratory markers including NLR for predicting the two disease courses of simple recovery and development of GI bleeding. In the ROC curve for simple recovery, the area under the ROC curve (AUROC) of NLR was 78.5% (95% confidence interval [CI], 64.7–92.2%). The optimal cut-off value determined using the Youden index was 3.18, and the sensitivity and specificity of NLR were 74.1% and 75.0%, respectively. In the ROC curve for GI bleeding, the AUROC of NLR was 88.6% (95% CI, 71.8–100.0%), and the optimal cut-off value was 3.90. Using this cut-off value, the sensitivity and specificity were 87.5% and 88.6%, respectively. On the contrary, MPV, ESR, and CRP were not useful for predicting simple recovery (AUROC [95% CI]: MPV, 39.6% [22.4–56.8%]; ESR, 42.9% [23.5–62.4%]; CRP, 47.6% [28.6–66.5%]). Additionally, those laboratory markers were less useful for predicting GI bleeding than NLR (AUROC [95% CI]: MPV, 39.8% [16.2–63.5%]; ESR, 44.3% [15.7–72.9%]; CRP, 59.8% [38.2–81.5%]).

**Fig 3:**
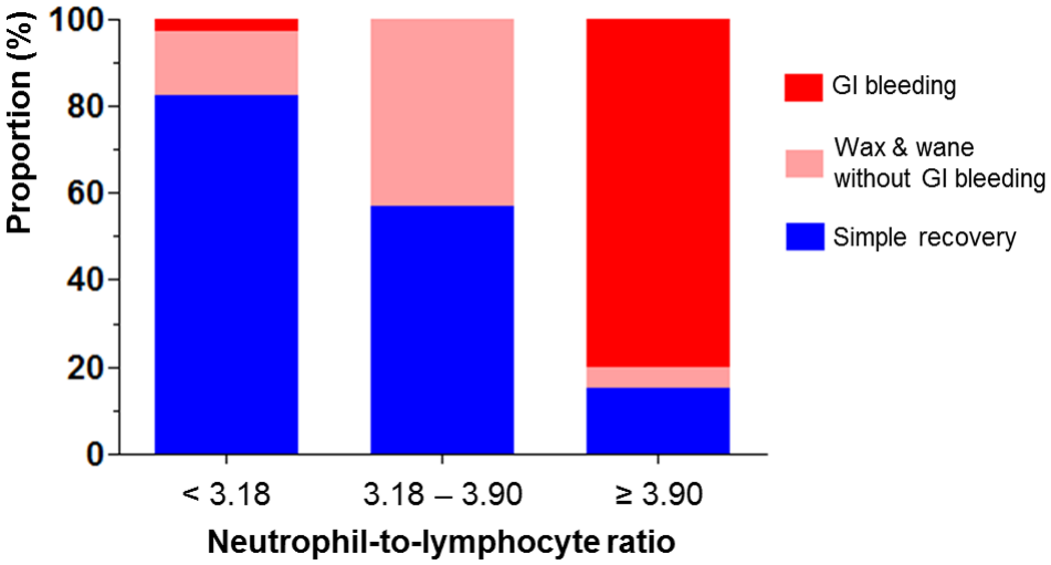
Clinical course of patients according to the two cut-off values of neutrophil-to-lymphocyte ratio. GI, gastrointestinal.

For the purpose of clinical application, we categorized the study population according to the two cut-off values of NLR (Fig 3). In patients with a low NLR (<3.18), 82.4% recovered easily without serious complication. However, in patients with a moderate NLR (3.18–3.90), 42.9% suffered from symptom relapse. In cases of patients with a high NLR (≥3.90), 80.0% showed GI bleeding.

### Clinical outcomes of patients with GI bleeding

**Fig 4:**
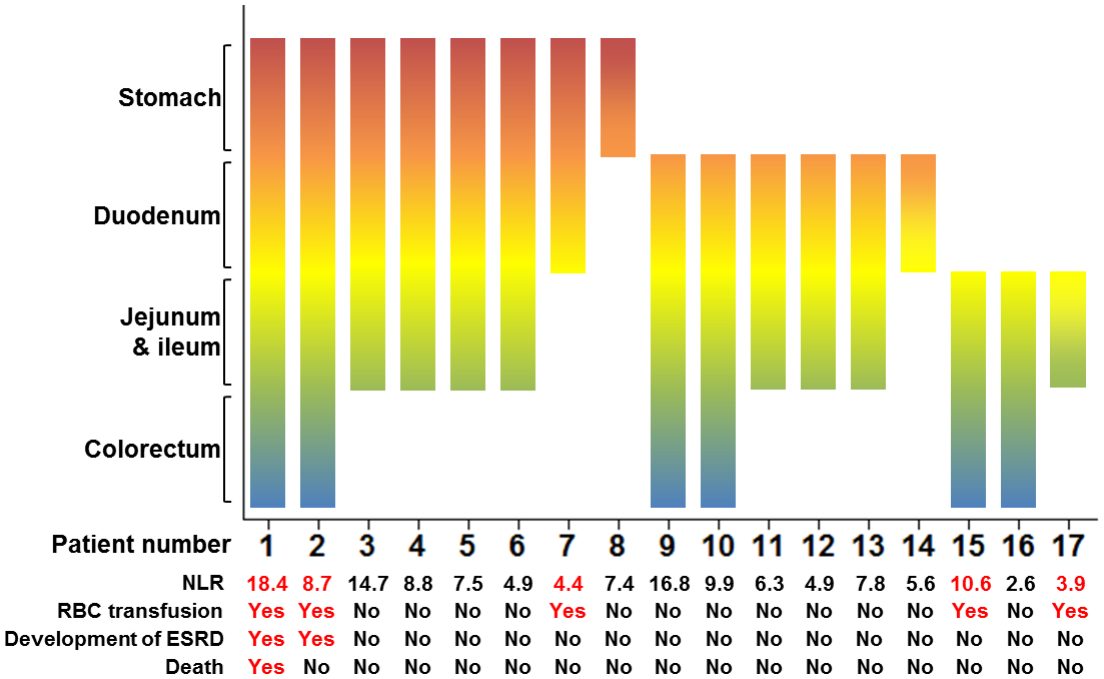
Clinical outcomes of patients with gastrointestinal bleeding. Bar represents the location of gastrointestinal bleeding in each patient. NLR, neutrophil-to-lymphocyte ratio; RBC, red blood cells; ESRD, end-stage renal disease.

In Fig 4, the detailed clinical outcomes of 17 patients with GI bleeding are presented. GI bleeding from the stomach or duodenum was identified in 14 patients (82.4%). Jejunal or ileal involvement of HSP was also common (82.4%). Colorectal involvement of HSP was relatively uncommon compared to upper GI tract (35.3%) involvement. Four of six patients with colorectal involvement had accompanying upper GI involvement of HSP. Of the 17 patients with GI bleeding, four required RBC transfusion. ESRD developed in the two patients who showed GI bleeding from the whole GI tract.

## Discussion

Although HSP is a systemic vasculitis that occurs predominantly in children [20], HSP can be of greater concern in adult patients than in pediatric patients because the prognosis is poorer in adults [2,21,22]. Adult patients with HSP have more serious GI bleeding and poorer renal prognosis [1–3,14,15]. Our study showed that GI bleeding developed in about 25% of patients. Additionally, 11.8% of patients with GI bleeding (2 of 17) suffered from ESRD. Because all of the patients without GI bleeding fully recovered without serious complications such as ESRD, we think that the development of GI bleeding is not only a significant manifestation of HSP, but also a barometer of overall disease status and prognosis. Therefore, we aimed to identify a laboratory marker for predicting GI bleeding in adult patients with HSP.

In this study, we focused on the NLR, which can be calculated simply using neutrophil and lymphocyte counts, because it is a well-evaluated laboratory marker of various adult diseases associated with inflammation [16–19]. In addition, a previous study has already demonstrated that the NLR is associated with GI bleeding in pediatric patients with HSP [11]. In that study, the optimal cut-off value for predicting GI bleeding was 2.82, and the sensitivity and specificity were 81.0% and 76%, respectively. Compared to the results for pediatric patients, the predictive performance of the NLR seemed to be superior in adult patients. Our study showed that the optimal cut-off value for predicting GI bleeding was 3.90, and that the sensitivity and specificity were 87.5% and 88.6%, respectively. Moreover, we determined that the NLR is also useful in predicting patient recovery without symptom relapse. For the purpose of predicting recovery without symptom relapse, the optimal cut-off value was 3.18. Using this cut-off value, the sensitivity and specificity for predicting a simple recovery were 74.1% and 75.0%, respectively. Therefore, the disease course in adult patients with HSP can be predicted using these two cut-off values, 3.18 and 3.90. If a patient shows a low NLR (<3.18), the risk of GI bleeding may be low (less than 3%). Additionally, most of these patients (more than 80%) will recover without symptom relapse. When a patient shows a moderate NLR (3.18–3.90), the risk of GI bleeding may be still low. However, a significant risk of symptom relapse (about 40%) is expected in these patients. Finally, if a patient shows a high NLR (≥3.90), the risk of GI bleeding increases up to about 80%. Only about 15% of these patients will recover without symptom relapse or GI bleeding.

Our model for predicting disease course can be useful in clinical practice, because the clinical symptoms and signs of GI bleeding may be delayed. Although the treatment method in our study was selected at the discretion of the clinician, it may depend on potentially-life threatening diseases, such as GI bleeding. Only 28.6% of patients (2 of 7) who showed GI bleeding before visiting the hospital were treated with supportive care, while 50.0% of patients (5 of 10) who showed GI bleeding after visiting the hospital received supportive care. If we can anticipate GI bleeding even though there are no initial symptoms or signs, early corticosteroid treatment can be administered.

Additionally, our data may provide a diagnostic strategy for evaluating GI bleeding. One of the interesting findings of our study is that upper GI involvement was common in patients who showed GI bleeding. Fourteen of 17 patients (82.4%) who showed GI bleeding had bleeding focused in the stomach or duodenum. Additionally, a considerable proportion of patients (66.7%) who showed colorectal involvement had lesions in the stomach or duodenum. These results imply that EGD should be considered in the initial workup of GI bleeding. Based on the data, we recommend EGD as an initial choice for GI bleeding evaluation in patients who show melena or hematemesis. Additionally, in the case of patients who show hematochezia, both EGD and colonoscopy are recommended, because there may be gastric and duodenal involvements as well as colorectal involvement.

Although this study was the first to evaluate the usefulness of the NLR for predicting disease course in adult patients with HSP, it has several limitations. The retrospective design of the study is the first limitation, as we could not control the treatment method used in the study population. Patients who showed GI and renal involvement tended to be treated with aggressive method such as steroid pulse therapy. Although we think that the poor prognosis of these patients may have been due to the severe disease manifestation rather than the steroid pulse therapy, the possible effects of different treatment modalities on prognosis should not be ignored. The small sample size is another limitation. Although we identified the association between NLR and disease course in HSP patients, external validation is mandatory to provide a definitive conclusion and establish a widely acceptable cut-off value of NLR.

Despite these limitations, our data provide a better understanding of the disease course of adult patients with HSP and the utility of NLR as a prognostic marker. The NLR is useful for predicting development of GI bleeding as well as simple recovery without symptom relapse. Two different cut-off values of NLR, 3.18 for predicting an easy recovery without symptom relapse and 3.90 for predicting GI bleeding can be used in adult patients with HSP.

## References

[1] Blanco R, Martinez-Taboada VM, Rodriguez-Valverde V, Garcia-Fuentes M, Gonzalez-Gay MA. Henoch-Schonlein purpura in adulthood and childhood: two different expressions of the same syndrome. Arthritis Rheum. 1997;40: 859–864. 9153547 10.1002/art.1780400513

[2] Pillebout E, Thervet E, Hill G, Alberti C, Vanhille P, Nochy D. Henoch-Schonlein Purpura in adults: outcome and prognostic factors. J Am Soc Nephrol. 2002;13: 1271–1278. 11961015 10.1097/01.asn.0000013883.99976.22

[3] Michel BA, Hunder GG, Bloch DA, Calabrese LH. Hypersensitivity vasculitis and Henoch-Schonlein purpura: a comparison between the 2 disorders. J Rheumatol. 1992;19: 721–728. 1613701

[4] Jennette JC, Falk RJ, Andrassy K, Bacon PA, Churg J, Gross WL, et al Nomenclature of systemic vasculitides. Proposal of an international consensus conference. Arthritis Rheum. 1994;37: 187–192. 8129773 10.1002/art.1780370206

[5] Nam EJ, Kim GW, Kang JW, Im CH, Jeon SW, Cho CM, et al Gastrointestinal bleeding in adult patients with Henoch-Schonlein purpura. Endoscopy. 2014;46: 981–986. 10.1055/s-0034-1377757 25321618

[6] Martinez-Frontanilla LA, Haase GM, Ernster JA, Bailey WC. Surgical complications in Henoch-Schonlein Purpura. J Pediatr Surg. 1984;19: 434–436. 6481588 10.1016/s0022-3468(84)80269-9

[7] Ronkainen J, Koskimies O, Ala-Houhala M, Antikainen M, Merenmies J, Rajantie J, et al Early prednisone therapy in Henoch-Schonlein purpura: a randomized, double-blind, placebo-controlled trial. J Pediatr. 2006;149: 241–247. 16887443 10.1016/j.jpeds.2006.03.024

[8] Weiss PF, Klink AJ, Localio R, Hall M, Hexem K, Burnham JM, et al Corticosteroids may improve clinical outcomes during hospitalization for Henoch-Schonlein purpura. Pediatrics. 2010;126: 674–681. 10.1542/peds.2009-3348 20855386 PMC3518383

[9] Jauhola O, Ronkainen J, Koskimies O, Ala-Houhala M, Arikoski P, Holtta T, et al Outcome of Henoch-Schonlein purpura 8 years after treatment with a placebo or prednisone at disease onset. Pediatr Nephrol. 2012;27: 933–939. 10.1007/s00467-012-2106-z 22311342

[10] Hong J, Yang HR. Laboratory markers indicating gastrointestinal involvement of henoch-schonlein purpura in children. Pediatr Gastroenterol Hepatol Nutr. 2015;18: 39–47. 10.5223/pghn.2015.18.1.39 25866732 PMC4391999

[11] Makay B, Gucenmez OA, Duman M, Unsal E. The relationship of neutrophil-to-lymphocyte ratio with gastrointestinal bleeding in Henoch-Schonlein purpura. Rheumatol Int. 2014;34: 1323–1327. 10.1007/s00296-014-2986-2 24647793

[12] Lin SJ, Huang JL, Hsieh KH. Clinical and laboratory correlation of acute Henoch-Schonlein purpura in children. Zhonghua Min Guo Xiao Er Ke Yi Xue Hui Za Zhi. 1998;39: 94–98. 9599897

[13] Saulsbury FT, Kesler RW. Thrombocytosis in Henoch-Schonlein purpura. Clin Pediatr (Phila). 1983;22: 185–187. 6825362 10.1177/000992288302200305

[14] Rieu P, Noel LH. Henoch-Schonlein nephritis in children and adults. Morphological features and clinicopathological correlations. Ann Med Interne (Paris). 1999;150: 151–159. 10392264

[15] Tancrede-Bohin E, Ochonisky S, Vignon-Pennamen MD, Flageul B, Morel P, Rybojad M. Schonlein-Henoch purpura in adult patients. Predictive factors for IgA glomerulonephritis in a retrospective study of 57 cases. Arch Dermatol. 1997;133: 438–442. 9126006 10.1001/archderm.133.4.438

[16] Proctor MJ, McMillan DC, Morrison DS, Fletcher CD, Horgan PG, Clarke SJ. A derived neutrophil to lymphocyte ratio predicts survival in patients with cancer. Br J Cancer. 2012;107: 695–699. 10.1038/bjc.2012.292 22828611 PMC3419948

[17] Balta S, Demirkol S, Unlu M, Arslan Z, Celik T. Neutrophil to lymphocyte ratio may be predict of mortality in all conditions. Br J Cancer. 2013;109: 3125–3126. 10.1038/bjc.2013.598 24084765 PMC3859933

[18] Kwon JH, Jang JW, Kim YW, Lee SW, Nam SW, Jaegal D, et al The usefulness of C-reactive protein and neutrophil-to-lymphocyte ratio for predicting the outcome in hospitalized patients with liver cirrhosis. BMC Gastroenterol. 2015;15: 146 10.1186/s12876-015-0378-z 26498833 PMC4619077

[19] Turkmen K, Guney I, Yerlikaya FH, Tonbul HZ. The relationship between neutrophil-to-lymphocyte ratio and inflammation in end-stage renal disease patients. Ren Fail. 2012;34: 155–159. 10.3109/0886022X.2011.641514 22172001

[20] Mills JA, Michel BA, Bloch DA, Calabrese LH, Hunder GG, Arend WP, et al The American College of Rheumatology 1990 criteria for the classification of Henoch-Schonlein purpura. Arthritis Rheum. 1990;33: 1114–1121. 2202310 10.1002/art.1780330809

[21] Kellerman PS. Henoch-Schonlein purpura in adults. Am J Kidney Dis. 2006;48: 1009–1016. 17162160 10.1053/j.ajkd.2006.08.031

[22] Uppal SS, Hussain MA, Al-Raqum HA, Nampoory MR, Al-Saeid K, Al-Assousi A, et al Henoch-Schonlein's purpura in adults versus children/adolescents: A comparative study. Clin Exp Rheumatol. 2006;24: S26–30. 16859592

